# From Inflammatory RNAs to Therapeutic Silencing: Deciphering the RNA–Inflammation Axis in Cancer and Neurodegeneration

**DOI:** 10.3390/biology15141106

**Published:** 2026-07-09

**Authors:** Emily Do, Durga Puro, Surajit Hansda

**Affiliations:** 1 Texas Tech University Health Sciences Center, Lubbock, TX 79430, USA; emily.do@ttuhsc.edu; 2 Department of Pharmaceutical Sciences, Jerry H. Hodge School of Pharmacy, Texas Tech University Health Sciences Center, ARB 2100A, 1406 South Coulter Street, Amarillo, TX 79106, USA; durpuro@ttuhsc.edu

**Keywords:** RNA–inflammation axis, non-coding RNAs, cancer inflammation, neuroinflammation, RNA therapeutics, neurodegenerative diseases, microRNAs

## Abstract

Inflammation is a critical protective response that maintains tissue homeostasis. However, persistent or dysregulated inflammation contributes significantly to the progression of cancer and neurodegenerative diseases. Recent advances in RNA biology have identified non-coding RNAs (ncRNAs), including microRNAs, long non-coding RNAs, and circular RNAs, as key modulators of inflammatory signaling networks. These RNA molecules regulate key pathways such as NF-κB, STAT3, MAPK, and PI3K/AKT, thereby influencing immune responses, tumor progression, neuronal survival, and cellular stress adaptation. In parallel, RNA-sensing receptors, including Toll-like receptors and RIG-I-like receptors, connect innate immune activation with chronic inflammatory pathology. Emerging evidence further demonstrates that inflammatory RNAs participate in epigenetic regulation, intercellular communication, and inter-organ crosstalk through extracellular vesicles and exosomes. In cancer, RNA-mediated feedback loops sustain tumor-promoting inflammation, metastasis, and immune evasion, whereas in neurodegenerative disorders, they contribute to glial activation, neuronal dysfunction, and progressive neuroinflammation. This review examines the mechanistic relationship between RNA dysregulation and inflammation across cancer and neurodegeneration, with particular emphasis on RNA signaling networks, exosomal communication, and targeted RNA-based therapeutics. Collectively, advances in understanding the RNA–inflammation axis may reveal new opportunities for precision diagnostics and next generation therapeutic interventions.

## Simple Summary

Chronic inflammation is now recognized as a major factor in the development of cancer and neurodegenerative diseases such as Alzheimer’s disease, Parkinson’s disease, and amyotrophic lateral sclerosis (ALS). Studies in RNA biology already have revealed that certain RNA molecules, once thought to function primarily in protein production, also play critical roles in initiating, amplifying, and sustaining inflammatory responses. This review explores how these inflammatory RNA molecules contribute to disease progression by regulating immune responses, cell survival, tumor growth, and brain inflammation. It further explores how damaged cells and tumors release small extracellular RNA-containing particles that can travel through the body and affect distant organs, including the brain. In addition, the review highlights emerging RNA-based therapies designed to block harmful inflammatory signals and improve treatment precision. Although significant challenges remain, particularly in delivering these therapies safely to target tissues such as the brain, advances in nanotechnology and biomimetic delivery systems are creating new opportunities for treatment. Understanding the relationship between RNA and inflammation may lead to earlier diagnosis, more personalized therapies, and improved outcomes for patients with cancer and neurodegenerative diseases, ultimately benefiting public health and quality of life.

## 1. Introduction

Inflammation is a fundamental protective response that enables the body to eliminate pathogens and repair damaged tissue. However, chronic or dysregulated inflammatory responses often contribute to the development of complex diseases, including cancer and neurodegenerative disorders. Increasing evidence over the past decade has positioned RNA, particularly non-coding RNA (ncRNA), at the center of inflammatory regulation. This emerging framework, often referred to as the RNA–inflammation axis, highlights the importance of RNA molecule function as not only intermediates of gene expression but also as active regulators and drivers of immune signaling and disease progression [1,2]. At the mechanistic level, inflammatory RNAs act as both initiators and modulators of innate immune responses. Pattern recognition receptors (PRRs), including Toll-like receptors (TLRs), RIG-I-like receptors (RLRs), and NOD-like receptors (NLRs), detect RNA molecules derived from pathogens or damaged host cells. Recognition of RNA by these receptors activates downstream signaling cascades, including NF-κB, MAPK, and interferon pathways, leading to the production of pro-inflammatory cytokines and chemokines [3,4]. While this process is essential for host defense, persistent activation of RNA-sensing pathways promotes chronic inflammation, thereby linking innate immunity to tumorigenesis and neurodegeneration [4,5].

Non-coding RNAs, including microRNAs (miRNAs), long non-coding RNAs (lncRNAs), and circular RNAs (circRNAs), play a central role in fine-tuning these inflammatory pathways. These molecules regulate gene expression at multiple levels and influence critical signaling networks such as PI3K/AKT/mTOR, Wnt/β-catenin, and MAPK pathways [1]. Dysregulation of ncRNAs has been widely reported in inflammatory diseases and cancers, where they can function either as tumor suppressors or oncogenes depending on the cellular context. For example, tumor-suppressive miRNAs such as the let-7 family and miR-34 are frequently downregulated in cancers, resulting in enhanced oncogenic signaling and uncontrolled cell proliferation [1]. Conversely, pro-inflammatory ncRNAs can amplify cytokine production and sustain a tumor-promoting microenvironment, reinforcing the link between chronic inflammation and cancer progression [1,6].

RNA sensing through TLRs represents a critical interface between RNA biology and innate immunity. Endosomal TLRs, including TLR3, TLR7, and TLR8, recognize double-stranded and single-stranded RNA species and initiate signaling through adaptor proteins such as myeloid differentiation primary response gene 88 (MYD88) and TIR-domain-containing adapter-inducing interferon (TRIF), ultimately activating NF-κB and interferon regulatory factors [4]. Importantly, these receptors respond not only to pathogen-associated molecular patterns (PAMPs) but also to endogenous danger-associated molecular patterns (DAMPs), including RNA released from damaged or dying cells [5]. This dual recognition mechanism allows RNA to act as a bridge between infection-induced and sterile inflammation. However, excessive or prolonged activation of RNA-sensing pathways contributes to chronic inflammatory conditions commonly observed in cancer and neurodegenerative diseases [4,5]. In addition to post-transcriptional regulation, RNA molecules also participate in epigenetic control of gene expression. RNA interference (RNAi) pathways, mediated by small RNAs such as siRNAs and piRNAs, guide Argonaute protein complexes to specific RNA targets, resulting in mRNA degradation or translational repression [7,8]. Beyond cytoplasmic silencing, RNAi mechanisms extend into the nucleus, where they direct chromatin modifications such as DNA methylation and histone modifications, leading to transcriptional gene silencing [7]. Studies in model organisms such as Schizosaccharomyces pombe have demonstrated that RNA-induced transcriptional silencing complexes (RITS) coordinate heterochromatin formation through histone H3K9 methylation, establishing stable gene repression [7]. Collectively, these epigenetic RNA-mediated mechanisms are increasingly recognized as important regulators of inflammatory gene expression and cellular identity.

A defining characteristic of the RNA–inflammation axis is interconnected feedback loops that sustain and amplify inflammatory signaling. Among these, the NF-κB pathway serves as a central regulatory hub. NF-κB controls the transcription of numerous inflammatory genes, including cytokines such as IL-6 and TNF-α, while also being regulated by ncRNAs [9]. This bidirectional interaction creates complex regulatory circuits. For instance, NF-κB-induced expression of Lin28B suppresses the let-7 family, leading to increased IL-6 production and activation of STAT3 signaling, which in turn reinforces NF-κB activity, thereby establishing a self-reinforcing inflammatory circuit [10]. Similar RNA-mediated feedback networks have been described in multiple cancers, where they contribute to tumor growth, metastasis, and immune evasion [6].

The pathological relevance of this axis is evident across diverse disease contexts. In cancer, chronic inflammation driven by ncRNA dysregulation plays a key role in shaping the tumor microenvironment. In breast cancer, for example, inflammatory signaling networks regulated by miRNAs and lncRNAs contribute significantly to tumor progression and metastasis, with approximately 35% of breast cancer-related deaths linked to deregulated inflammation [6]. In brain tumors such as glioblastoma, ncRNAs regulate critical processes including proliferation, angiogenesis, and immune evasion through interactions with major signaling pathways [11,12]. Similarly, in lung cancer, ncRNAs modulate epithelial–mesenchymal transition (EMT), metastasis, and therapeutic resistance by regulating pathways such as TGF-β signaling [9,10]. Beyond oncology, the RNA–inflammation axis is equally critical in neurodegenerative diseases. Dysregulated ncRNA expression has been implicated in conditions such as Alzheimer’s disease (AD), Parkinson’s disease (PD), and amyotrophic lateral sclerosis (ALS), where it contributes to neuroinflammation and progressive neuronal loss [6,13]. In these disorders, ncRNAs regulate the activation states of microglia and astrocytes, influencing the balance between neurotoxic and neuroprotective responses. For example, miR-146a modulates inflammatory signaling by suppressing IL-6 and COX-2 expression, while lncRNAs such as SNHG1 and lncRNA-p21 regulate microglial activation through complex feedback interactions [6]. In ALS, dysfunction of RNA-binding proteins such as TDP-43 and FUS leads to widespread disruption of RNA processing, coupled with chronic inflammatory signaling, creating a self-reinforcing cycle of neurodegeneration [13].

Collectively, these findings support a unifying concept that inflammatory RNAs are not merely passive biomarkers but active drivers of disease across tissues and organ systems. Despite significant progress, important questions remain regarding how RNA-mediated inflammatory networks are coordinated, how they transition from protective to pathological states, and how they can be therapeutically targeted with precision. In this review, we aim to integrate current knowledge on the RNA–inflammation axis, from mechanistic insights and regulatory feedback loops to inter-organ communication and therapeutic strategies. By bridging RNA biology with inflammation-driven pathology in cancer and neurodegeneration, this work highlights the translational potential of targeting inflammatory RNAs for next-generation precision therapies.

In this review, we define the RNA–inflammation axis as a bidirectional molecular network in which both coding and non-coding RNAs regulate inflammatory signaling pathways, while inflammatory stimuli, in turn, influence RNA transcription, processing, stability, and intercellular transfer. This dynamic interaction forms self-sustaining feedback loops involving NF-κB, STAT3, inflammasome signaling, and RNA-sensing receptors that contribute to chronic inflammation, tumor progression, and neurodegeneration. Understanding this axis provides a unifying framework for identifying common pathogenic mechanisms and developing RNA-based therapeutic strategies.

## 2. The Mechanistic Link: Inflammatory RNAs as Pathological Drivers

### 2.1. Non-Coding RNAs (ncRNAs)

Inflammation is a protective physiological response that enables the body to recognize and eliminate harmful stimuli. However, various autoimmune diseases, sepsis, and other chronic inflammatory disorders have been shown to arise from chronic or dysregulated inflammatory responses [14]. A key component of innate immunity, the body’s first line of defense, is the inflammasome, a multiprotein complex assembled from PRRs, including NLRs, AIM2, and pyrin [14]. These receptors function as molecular sensors that recognize cellular damage, stress signals, and invading pathogens. Beyond canonical immune-sensing mechanisms, increasing evidence highlights ncRNAs as important regulators of inflammatory pathology. Their regulatory effects are closely tied to their biogenesis and structural features, which determine how they function in cellular signaling networks [15].

A ncRNA can contribute to chronic inflammation and cancer progression. Depending on the circumstances, ncRNAs can act as tumor suppressors or oncogenes. In inflammatory conditions, ncRNAs regulate the production of pro-inflammatory mediators, such as cytokines and chemokines, that help maintain the tumor-promoting microenvironment. The continuous inflammation can aid in the tumor’s growth, progression, and metastasis [14]. In addition, long-term inflammation caused by infections or immune system problems can lead to cellular changes that increase the risk of cancer. Because ncRNAs are often tissue-specific, they can be useful biomarkers for treatment and disease monitoring.

### 2.2. RNA-Sensing TLRs (Toll-Like Receptors)

TLRs are a major class of PRRs that detect nucleic acids and initiate innate immune signaling. RNA, a single-stranded polymer found in living organisms, acts as an immunostimulatory signal in the innate immune system and is detected by PRRs. RNA-sensing TLRs are predominantly localized within endolysosomal compartments, where they monitor nucleic acid ligands while minimizing recognition of self-RNA [16]. Humans express approximately ten TLR family members, each composed of an extracellular leucine-rich repeat (LRR0 domain), a transmembrane segment, and an intracellular Toll/interleukin-1 receptor (TIR) signaling domain. The LRR domain is responsible for recognizing PAMPs, while the TIR initiates intracellular signaling by recruiting adaptor proteins. Although endosomal TLRs represent major RNA sensors, additional PRR families contribute to RNA-mediated immune surveillance [17].

Other types of RNA-sensing pattern recognition receptors, such as RLRs, detect viral RNA in the cytoplasm. RIG-I can recognize dsRNA with specific features such as blunt ends and 5′-triphosphate groups [16]. It can also recognize ssRNA, which contains specific viral features such as a 5′ triphosphate group [18]. These structural features allow RIG-I to be able to distinguish viral RNA from most cellular RNA that lacks these 5′ end characteristics. Melanoma differentiation-associated protein 5 (MDA-5) is another member of the RLR family and recognizes positive-strand RNA viruses [19]. When MDA5 and RIG-I detect viral RNA in a cell, they activate the mitochondria antiviral signaling protein (MAVS), which initiates innate immune signaling. MAVS then activates downstream signaling molecules such as TBK1 and IKK, leading to activation of transcription factors interferon regulatory factor 3 (IRF3) and NF-kβ. These TF will trigger the production of type I interferons and other inflammatory molecules that help fight off the infection [20].

Another type of RNA-related immune sensor is NLRs, which are the largest group of PRRs in animals [16]. NLRs are also known to respond to RNA signals. An example is NLRP1, which is involved in inflammasome activation, a large protein complex that activates inflammatory responses. NLRP1 does not directly bind dsRNA but can be activated during viral infection [21].

When RNA is detected by these receptors, they activate different signaling pathways that lead to the production of inflammatory molecules. TLRs, for example, signal through adaptor proteins such as MYD88 and TRIF. This leads to the production of inflammatory cytokines. For this process to occur correctly, RNA-sensing TLRs must first be located in the endosomal compartment, where they are protected from the cell’s own RNA. The chaperone protein UNC93B1 is essential for transporting TLR3, TLR7, and TLR8 from the endoplasmic reticulum to the endosomes, allowing them to function properly in response to viral RNA [22].

TLR3 is a receptor that detects dsRNA, which is commonly produced during viral infections. In its inactive state, TLR3 is made of leucine-rich repeat domains. When dsRNA is present, it must bind between two TLR3 molecules and form a dimer. TLR7 and TLR8 detect single-stranded RNA (ssRNA), typically generated after RNA is degraded in endosomes. These receptors contain two binding sites: one for small RNA (single nucleosides) and another for short RNA (short oligonucleotides) fragments. Once these receptors recognize RNA and are properly localized by UNC93B1, they recruit adaptor proteins and trigger downstream signaling pathways that lead to inflammatory cytokine production [16].

TLRs play an important role in activating the innate immune system by recognizing markers expressed by PAMPs, but they can also recognize signals released by damaged or dying cells, known as DAMPs or alarmins. While they help alert the immune system, too many alarmins can lead to chronic inflammation, which is commonly seen in cancer. Many cancers, such as breast, colon, and pancreatic cancer, show increased levels of these signals because of ongoing cell damage and inflammation [23].

### 2.3. Epigenetic RNA Signaling

RNA molecules regulate gene expression not only through post-transcriptional mechanisms but also through epigenetic pathways that modify chromatin structure and transcriptional activity [8]. Different types of RNAs, such as small ncRNAs and lncRNAs, contribute to genome regulation and stability. Small RNAs can direct transcriptional regulation through the RNAi pathway. In these mechanisms, Argonaute containing complexes are guided by small RNAs to complementary RNA transcripts, which can facilitate the recruitment of chromatin modifying enzymes, including histone modifying enzymes and DNA methyltransferases. These modifications alter DNA’s chromatin structure and result in transcriptional repression. In addition, lncRNAs can also regulate gene expression independently of small RNAs by acting as scaffolds that recruit chromatin modifying complexes to specific genomic regions. Through these mechanisms, lncRNAs can influence chromatin structure without recruiting RNAi machinery [24]. Collectively, these RNA mediate processes contribute to transcriptional regulation by creating chromatin states that can persist over time. This allows cells to retain gene expression patterns in response to different signals. In contrast, RNAi pathways in the cytoplasm function at the post transcriptional level. An Argonaute protein with small RNA regulates gene expression by either degrading mRNA or preventing its translation into protein. In humans, Argonaute proteins can function both in the nucleus and in the cytoplasm, although their predominant role is post transcriptional regulation [8,25].

In many organisms, these small RNAs are produced from intergenic regions or antisense transcription and help regulate chromatin structure. RNAi pathways in the nucleus can direct DNA methylation and histone modifications, leading to transcriptional gene repression. This mechanism was first observed in *Arabidopsis thaliana* and has since been found in other organisms such as Caenorhabditis elegans and yeast, showing that RNAi plays a widespread role in gene silencing and heterochromatin formation [26,27]. First, a long dsRNA molecule is cut into shorter fragments called siRNAs. These siRNAs are then loaded onto Argonaute proteins. The siRNA acts like a guide, leading the AGO protein to match RNA in the cell. When the binding occurs, the gene can be silenced by breaking down the RNA, blocking protein production, or shutting the gene down by changing the DNA packaging [8].

The fission yeast *Schizosaccharomyces pombe* has emerged as a foundational model for understanding RNAi-mediated transcriptional gene silencing because its RNAi machinery is highly conserved and directly linked to heterochromatin assembly. In this organism, small RNAs guide silencing complexes to specific genomic regions, promoting histone H3 lysine 9 (H3K9) methylation and stable gene repression. Key RNAi genes, including *ago1+*, *dcr1+*, *and rdp1+*, are essential for this process, and deletion of these genes disrupts heterochromatin formation and transcriptional silencing [8,28].

## 3. The RNA–Inflammation Axis: Core Pathways and Feedback Loops

Inflammation is important for protecting organisms from foreign invaders. It depends on precise control of gene expression to balance pro-inflammatory and anti-inflammatory responses. While many mechanisms regulating acute inflammatory responses have been well characterized, the molecular processes that sustain chronic inflammation remain incompletely understood. Disruptions in gene regulation, including mutations and abnormalities in RNA processing, can contribute to persistent inflammation and diseases such as cancer [29]. This protective process involves the coordinated actions of chemokines, cytokines, and various other inflammatory cells to eliminate damaging agents and promote tissue repair. Persistent inflammation is strongly associated with conditions such as asthma, diabetic retinopathy, and PD [30]. Key inflammatory mediators include cytokines such as IL-1β, IL-6, IL-8, IL-10, and TNF-α, which play a significant role in disease progression. Despite these associations, the mechanisms that regulate inflammation remain unknown [30].

An emerging layer of regulation involves lncRNAs, which are recognized as important regulators in the inflammatory response by their ability to control gene transcription. They can interact with DNA, RNA, and proteins to influence cellular processes. lncRNAs can act as signals, scaffolds, or guides, each contributing to the regulation of gene expression. Because of this, lncRNAs can participate in chromatin remodeling, RNA processing, and mRNA degradation [30]. In addition, some lncRNAs function as competing endogenous RNAs (ceRNAs) acting as miRNA sponges that bind microRNA and regulate the expression of their target genes.

At the upstream end of the RNA–inflammation axis, the innate immune system relies on the receptors to recognize harmful microbes. These receptors detect structures found on pathogens but not in the human body. These are known as PAMPs. PAMPs are essential for pathogen survival, so they do not readily change, making them reliable targets for the immune system. PAMPs are detected by PRRs. There are numerous PRRs in the human body, such as TLRs, Rig-I-like receptors, AIM2-like receptors (ALRs), and others. When these receptors detect PAMPs, they trigger an immune response, including inflammation and the release of cytokines and chemokines [29].

A central downstream hub linking RNA-mediated sensing to inflammatory gene expression is NF-κB, a family of transcription factors that help to regulate the expression of genes involved in inflammation. One way they do this is by working with miRNAs that regulate gene expression at the post-transcriptional level. NF-κB is composed of five main members, including p65 (RelA), RelB, p50, p52, and c-rel, which regulate gene expression by binding to kB DNA elements as dimers. In the resting state, NF-κB is inactive in the cytoplasm due to inhibitory proteins, especially IκBα. Activation of the canonical NF-κB pathway begins when inflammatory cytokines or pathogen-associated molecular patterns bind to receptors such as TNF receptors, IL-1 receptors, or TLRs. These receptors recruit adaptor proteins including MyD88, TRIF, tumor necrosis factor receptor-associated factor 6 (TRAF6), and receptor-interacting serine/threonine-protein kinase 1 (RIPK1), leading to activation of the IκB kinase (IKK) complex. Activated IKK phosphorylates IκBα, targeting it for ubiquitination and proteasomal degradation, thereby releasing NF-κB to translocate into the nucleus and induce the transcription of inflammatory genes [31]. The canonical pathway is activated by cytokines and PRRs, primarily activating p50/RelA and p50/c-Rel. In contrast, the non-canonical pathway is slower and depends on NF-κB inducing kinase (NIK), which activates IKKα and promotes the processing of p100 into p52. This leads to the formation of the p52/RelB complex, which regulates a subset of immune related genes [32].

The NF-κB, STAT3, and MAPK pathways are not independent signaling modules but form a highly interconnected regulatory network that sustains chronic inflammation. Activation of NF-κB induces the production of pro-inflammatory cytokines such as IL-6 and TNF-α. These secreted cytokines subsequently bind to their cognate receptors on the same cell or neighboring cells, leading to activation of Janus kinases (JAKs), phosphorylation of STAT3, and STAT3 dimerization and nuclear translocation, where it regulates the transcription of genes involved in inflammation, cell survival, proliferation, and immune responses. Activated STAT3 further cooperates with NF-κB to maintain persistent inflammatory signaling and establish self-reinforcing feedback loops [33]. Activated STAT3, in turn, promotes the expression of additional inflammatory mediators and cooperates with NF-κB to maintain a persistent inflammatory state. Simultaneously, the MAPK pathways, including ERK, JNK, and p38, are activated downstream of cytokine receptors, TLRs, and growth factor receptors through sequential phosphorylation cascades involving MAP3Ks and MAP2Ks. Activated MAPKs stimulate transcription factors such as AP-1 and cooperate with NF-κB to enhance the expression of pro-inflammatory cytokines, including IL-1β, IL-6, and TNF-α. These cytokines further activate JAK/STAT3 signaling in autocrine and paracrine manners [34]. Non-coding RNAs contribute to these signaling loops by modulating the expression of upstream receptors, transcription factors, and cytokines, thereby establishing self-reinforcing feedback circuits that drive tumor progression and neuroinflammation. Disruption of these regulatory networks can result in sustained inflammation and disease progression [35,36].

NF-κB can control the production of miRNAs, and in turn, miRNAs can regulate NF-κB activity. This creates a feedback loop and a complex network that helps control how long inflammatory responses last and determines the cell’s fate. Several miRNAs participate in both positive and negative feedback loops that regulate inflammatory signaling. Among them are miR-155 and miR-146a. Both are transcriptionally induced by NF-κB [37]. miR-146a functions as a negative regulator of inflammation by suppressing NF-κB and AP-1 signaling, helping shut down the inflammatory response and prevent excessive cytokine production. In contrast, miR-155 promotes immune cell activation and inflammatory signaling [38]. The NF-κB family of transcription factors plays a central role in regulating inflammatory and stress responses.

NF-κB controls many genes involved in inflammation, immune responses, and cell survival. As a result, it has a wide range of effects that depend on the cell type and conditions. In cancer, NF-κB often plays a tumor-promoting role. It helps cancer cells survive, grow, and spread. It also changes the tumor microenvironment by increasing inflammatory cytokines. Under normal conditions, NF-κB activity is tightly controlled by negative feedback loops, but in cancer cells, these control systems often fail, leading to constant NF-κB activation [39]. When activated, it increases the production of inflammatory molecules such as IL-6, thereby promoting tumor growth and altering the tumor microenvironment. These signals can also be activated by other pathways, such as STAT3, further supporting cancer progression, specifically, breast and lung cancer [39]. NF-κB interacts with other signaling pathways, such as p53 and STAT3, which further influence tumor development. In addition, NF-κB promotes processes such as epithelial-to-mesenchymal transition (EMT), which promotes cancer cell spread, and stimulates blood vessel formation via factors such as VEGF [39,40]. Together, these interconnected RNA- and NF-κB-driven feedback loops provide a mechanistic framework for how dysregulated RNA signaling can lock inflammation into a persistent state that fuels both cancer progression and chronic inflammatory disease.

## 4. Disease-Specific Feedback Loops

miRNAs are among the most extensively studied non-coding RNAs in cancer and inflammation due to their widespread dysregulation and strong impact on cellular signaling networks. Abnormal miRNA expression is observed in many human cancers, where it disrupts key inflammatory and immune-related pathways that regulate cell proliferation, apoptosis, migration, and immune responses. miRNAs have various roles, including acting as tumor suppressors by preventing uncontrolled cell growth. For example, Let-7 functions as a tumor suppressor miRNA that is frequently downregulated in cervical, breast, and lung cancer, leading to reduced inhibition of the RAS oncogene family. miR-15a/miR-16-1 is also often found at low levels in chronic lymphocytic leukemia (CLL) [15]. Another tumor suppressor family that works with the p53 tumor suppressor pathway is the miR-34 family. The miR-34 family helps stop a process called epithelial–mesenchymal transition (EMT), which makes cancer cells more mobile. Low levels of miR-34 have been found in several cancers, such as prostate, breast, bone, blood, and gastrointestinal [15].

### 4.1. Breast Cancer

Breast cancer remains one of the leading causes of cancer related mortality among women worldwide [41]. Over the past few decades, increased awareness has led to improved screening, early detection, and better treatment options. However, breast cancer mortality remains high and continues to be a major health concern. Recent studies have highlighted the importance of non-coding RNAs in breast cancer. These regulatory RNAs help control gene expression and are involved in cancer development, progression, and metastasis [41]. Inflammation is a major driver of breast cancer progression, with approximately 35% of breast cancer related deaths linked to abnormal or deregulated inflammatory signaling [41]. The immune system and inflammatory processes within the breast tumor microenvironment play an important role in cancer recurrence and dormancy. Inflammation is additionally and important factor in breast cancer progression, especially in conditions such as obesity, where it can strongly affect the tumor environment. Recent research has focused on how ncRNAs regulate inflammation processes in tumors. One major pathway is the NF-κB signaling pathway, which plays a central role in breast cancer development and progression [41]. NF-κB is involved in tumor initiation, growth, and metastasis, making it an important therapeutic target. NF-κB regulates the expression of multiple cytokines and chemokines, which are critical mediators of inflammation within the tumor microenvironment [42]. Importantly, NF-κB also participates in key inflammatory regulatory circuit in which it induces Lin28B, leading to suppression of the Let-7 microRNA family. Since Let-7 normally represses IL-6 expression, its inhibition results in increased IL-6 production. IL-6 subsequently binds to the IL-6 receptor (IL-6R/gp130) on the same or neighboring cells, activating JAKs that phosphorylate STAT3, leading to its dimerization, nuclear translocation, and transcription of genes involved in inflammation, proliferation, and cell survival. IL-6/STAT3 signaling, in turn, reinforces NF-κB activity, forming a positive feedback loop that sustains chronic inflammation and promotes malignant transformation [43]. Evidence from miRNA-mediated regulatory networks further supports the existence of interconnected inflammatory signaling circuits in breast cancer. Multiple miRNAs have been shown to modulate NF-κB activity, IL-6/STAT3 signaling, and downstream cytokine expression, acting as either positive or negative regulators of inflammation in breast cancer. Collectively, these findings highlight the presence of complex RNA-mediated feedback circuits that integrate inflammatory signaling with tumor progression [41,44]. For example, miR-155 enhances breast cancer inflammation by directly suppressing SOCS1, a negative regulator of the JAK/STAT3 pathway, thereby prolonging STAT3 activation and increasing IL-6 production. Similarly, miR-1246 promotes a pro-inflammatory tumor microenvironment by targeting tumor suppressor genes such as CCNG2, leading to enhanced NF-κB activity and increased secretion of inflammatory cytokines, including IL-6, CCL2, and CCL5. In contrast, miR-520/373 and miR-146b function as negative regulators by suppressing NF-κB signaling and reducing IL-6 and IL-8 production, highlighting their tumor-suppressive roles in inflammation [41].

Not only are miRNAs connected to inflammation, but lncRNAs are another class of regulatory non-coding RNAs involved in breast cancer. Compared with microRNAs, the roles of lncRNAs are less well understood, but evidence still indicates that they contribute to inflammatory signaling and tumor progression. One example of a lncRNA is NKILA (NF-κB interacting LncRNA). NKILA is induced by NF-kB signaling and functions as a negative feedback regulator of the NF-κB pathway. It binds to the NF-kB/IkB complex and prevents phosphorylation of IkB, thereby reducing excessive activation of NF-kB during inflammatory responses [41,43].

### 4.2. Glioblastoma

The central nervous system (CNS) tumors are one of the deadliest and most difficult cancers to treat [45]. There are four grades of brain tumors based on their aggressiveness. Glioblastoma multiforme (GBM) is a grade four tumor that is the most common and accounts for at least 80% of primary brain cancers [45,46]. The brain is a common site for cancers to spread from other parts of the body. Nearly 40% of cancers outside the brain can eventually metastasize to the brain [20,45]. Brain cancer develops over time through multiple steps, starting with genetic changes in cells that are not cancerous yet. Many mutations have been identified in brain tumors, including genes like *tumor protein 53* (*TP53*), *phosphatase* and *tensin homolog* (*PTEN*), *neurofibromatosis type 1* (*NF1*), and others. Most of these genes normally act as tumor suppressors. Another important alteration observed in many brain cancers is the deletion of the *CDKN2A* (*p16*) locus. *p16* normally restrains CDK4/6-mediated G1-to-S phase cell-cycle progression by maintaining RB in its active state. Loss of *p16* removes this important cell-cycle checkpoint, facilitating uncontrolled proliferation when accompanied by additional oncogenic alterations, such as defects in the RB or TP53/p19ARF pathways [47]. These genetic mutations affect signaling pathways that regulate how cells behave. These mutations converge on several major oncogenic pathways, including RTK (receptor tyrosine kinases)/PI3K/RAS signaling, the p53 pathway, RB pathway, and IDH1/2-associated metabolic/epigenetic rewiring. In particular, mutant IDH1/2 generates D-2-hydroxyglutarate (D-2HG), which drives broad epigenetic changes and contributes to tumor development through HIF-1 activation and CpG island hypermethylation, including silencing of tumor suppressor genes.

LncRNAs play an important role in the development and progression of glioblastoma by controlling key cancer-related pathways such as Wnt/β-catenin, PI3K/Akt, p53, and inflammatory signaling networks [48,49]. In early tumor development, dysregulation of p53-associated lncRNAs is linked to increased expression of SOX TFs, thereby promoting tumor progression. The role of lncRNAs in glioma initiation is further supported by evidence that several tumors suppressive and oncogenic lncRNAs regulate signaling pathways involved in gliomagenesis. For example, CASC7 inhibits glioma formation and progression by suppressing the Wnt/β-catenin signaling, whereas CASC9, together with miR-519d and STAT3 [50], forms a positive feedback loop that promotes tumor growth and progression. Similarly, AGAP2-AS1 promotes glioma growth by blocking microRNAs and activating the Wnt/β-catenin pathway [51], while NEAT1 increases tumor progression by enhancing EGFR-dependent Wnt/Beta-catenin activation with interactions with EZH2 [52].

### 4.3. Lung Cancer

**Figure 1:**
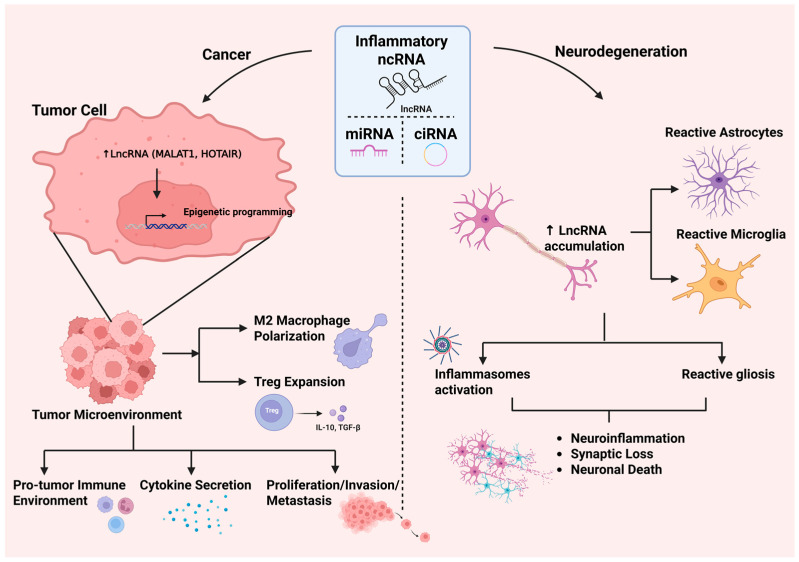
The ncRNA–inflammation axis: shared non-coding RNA classes drive divergent pathologies in cancer and neurodegeneration: Inflammatory non-coding RNAs (ncRNAs), including long non-coding RNAs (lncRNAs), microRNAs (miRNAs), and circular RNAs (circRNAs), act as shared upstream regulators of inflammatory gene expression programs in both oncological and neurodegenerative contexts. In cancer (**left**), overexpressed oncogenic lncRNAs (e.g., MALAT1) drive epigenetic reprogramming in the tumor cell nucleus by recruiting transcription factors and chromatin remodeling complexes. This reshapes the tumor microenvironment through M2 polarization and regulatory T cell (Treg) expansion, promoting secretion of immunosuppressive cytokines (IL-10, TGF-β) and pro-inflammatory mediators. The resulting pro-tumor immune microenvironment facilitates immune evasion, cytokine-driven inflammation, and ultimately tumor proliferation, invasion, and metastasis. In neurodegeneration (**right**), pathological accumulation of lncRNAs in neurons and glial cells activates innate immune signaling cascades, including NLRP3 inflammasome activation and reactive gliosis characterized by microglial and astrocyte reactivity. These converging inflammatory responses drive neuroinflammation, synaptic loss, and neuronal death, which are hallmarks of AD and PD pathology. **Abbreviations:** MALAT1: Metastasis-Associated Lung Adenocarcinoma Transcript 1; HOTAIR: HOX transcript antisense RNA; Treg: Regulatory T cells; IL-10: Interleukin-10; TGF-β: Transforming Growth Factor-beta.

Lung cancer is one of the most common and deadly cancers in the world, with about 2.2 million new cases and 1.8 million deaths in 2020 [53]. It often has high death rates because it is usually found late, grows quickly, and can come back after treatment. Even with improved tests and treatments, survival rates remain low. Recent research shows that ncRNAs, including miRNAs, lncRNAs, and cirRNAs, help regulate gene expression and play important roles in lung cancer by influencing how tumors initiate, grow, spread, and develop resistance to treatment (Figure 1). Some ncRNAs can act like oncogenes, while others act like tumor suppressors. They are involved in key cancer processes like epithelial–mesenchymal transition (EMT), which helps cancer spread to other parts of the body [53]. Beyond regulating metastasis, several lncRNAs directly modulate inflammatory signaling in lung cancer. MALAT1 promotes activation of the NF-κB pathway by functioning as a competing endogenous RNA for multiple anti-inflammatory miRNAs, resulting in increased expression of inflammatory cytokines, including IL-6 and TNF-α, and enhanced macrophage polarization that supports tumor progression [53]. Likewise, LCAT1 sponges miR-4715-5p, increasing RAC1 activity to promote tumor invasion, while inflammatory signaling through NF-κB and STAT3 further amplifies epithelial–mesenchymal transition and metastatic dissemination [53]. ncRNAs also interface with TGF-β signaling, a major EMT driver, by modulating EMT transcription factors such as SNAIL1/2, ZEB1/2, and TWIST, which repress E-cadherin and promote invasion and migration [54]. Beyond their roles in metastasis and EMT, several ncRNAs directly regulate inflammatory signaling in lung cancer. For example, miR-21 promotes tumor progression by activating NF-κB and STAT3 signaling and increasing IL-6 production, whereas lncRNA MALAT1 enhances inflammatory cytokine expression and macrophage polarization. Conversely, tumor suppressor miRNAs such as miR-146a negatively regulate NF-κB-dependent inflammation, highlighting the close association between RNA dysregulation and inflammatory tumor progression [55,56].

### 4.4. Neurodegenerative Diseases

In neurodegenerative diseases, ncRNAs are increasingly recognized as important regulators of progressive neuronal loss and functional decline in the CNS. Dysregulation of ncRNA expression has been reported in disorders such as AD, PD, and Huntington’s disease (HD). Neuroinflammation, defined as an inflammatory response within the central nervous system mediated by glial cells such as microglia and astrocytes, plays a critical role in the progression of these diseases [57]. Under normal conditions, neuroinflammation contributes to tissue repair and neuroprotection; however, excessive activation of glial cells leads to the overproduction of inflammatory mediators, including cytokines, chemokines, and ROS, which can further cause neuronal damage and promote neurodegeneration. NcRNAs play an important role in regulating these inflammatory processes (Figure 1). miRNAs influence inflammation by targeting messenger RNAs and controlling their stability or translation [24]. For example, miR-146a has been shown to reduce IL-6 and COX-2 expression, demonstrating its involvement in the nervous system [24].

An important aspect of neuroinflammation in neurodegenerative disease is the balance between harmful and protective states of microglia and astrocytes. These cells can adopt a neurotoxic or neuroprotective role of M1/A1 and M2/A2, respectively. Recent advances in single-cell RNA sequencing have revealed substantial heterogeneity among microglial and astrocyte populations during neurodegeneration. Disease-associated microglia (DAM) are characterized by increased expression of genes involved in phagocytosis, lipid metabolism, and inflammatory signaling, including APOE, TREM2, and CST7 [58,59]. Additional subsets such as interferon-responsive microglia (IRM), proliferative-region-associated microglia (PAM), and activated response microglia (ARM) exhibit distinct transcriptional programs associated with antiviral responses, proliferation, and chronic neuroinflammation. Similarly, astrocytes display diverse reactive states beyond the classical A1/A2 classification, with specific inflammatory and neuroprotective phenotypes identified through single-cell transcriptomic analyses [60,61]. Importantly, many of these cellular states are regulated by non-coding RNAs and inflammatory signaling pathways, further supporting the central role of the RNA–inflammation axis in neurodegenerative disease progression.

Neurotoxic cells release inflammatory factors (TNF-alpha, IL-16, CCL2-IL-18) that contribute to neuronal damage, while neuroprotective cells release factors (arginase 1, IGF-1, Fzd1) that support repair [24]. In neurodegenerative diseases, this balance is often disrupted, leading to increased inflammation, and ncRNAs help regulate this shift by influencing glial cell activity and its downstream consequences.

### 4.5. Parkinson’s Disease

In PD, lncRNA-p21 promotes microglial activation by sequestering miR-181, thereby increasing PKC-δ expression. Activated PKC-δ subsequently stimulates NF-κB and MAPK signaling pathways, leading to elevated production of TNF-α, IL-1β, and reactive oxygen species. These inflammatory mediators further enhance p53-dependent lncRNA-p21 expression, forming a positive feedback loop that sustains neuroinflammation and dopaminergic neuronal injury [62]. SNHG1 functions as a competing endogenous RNA that sequesters miR-7, thereby relieving repression of NLRP3 inflammasome-associated targets. Reduced miR-7 activity promotes microglial activation and NF-κB signaling, leading to increased production of pro-inflammatory cytokines and neuronal damage. This SNHG1/miR-7 axis represents an important RNA-mediated mechanism underlying chronic neuroinflammation in Parkinson’s disease [62,63].

### 4.6. Amyotrophic Lateral Sclerosis

ALS is recognized as the most common disorder affecting both upper and lower motor neurons. RNA expression is extensively disrupted in ALS due to dysfunction of key RNA-binding proteins, particularly TDP-43 and FUS, which regulate thousands of transcripts essential for neuronal function. TDP-43 normally functions in the nucleus to regulate RNA transcription, splicing, stability, and transport, but in ALS it becomes mislocalized to the cytoplasm, leading to both loss of normal function and toxic aggregation [64]. This results in widespread alteration in gene expression, including genes involved in synaptic activity, ion channel regulation, and neuronal maintenance. Mutations in FUS cause similar issues, disrupting gene expression in ways that affect neuron health. In addition, repeat expansions of *C9orf72* further alter gene expression, especially in pathways important for synapses and cell stability [64]. Problems with RNA editing, particularly involving ADAR2, also make motor neurons more vulnerable by affecting glutamate receptors and increasing the risk of excitotoxic damage. Overall, these changes show that disruption of RNA regulation can play a major role in causing neuronal degeneration in ALS.

ALS involves not only problems with RNA processing but also a connection between RNA dysfunction and inflammation in the nervous system. When RNA-binding proteins such as TDP-43 and FUS are disrupted, cells cannot properly manage stress, leading to abnormal stress granules and the buildup of toxic RNA-protein aggregates. These changes also interfere with immune signaling in the brain, causing an imbalance in inflammatory molecules such as cytokines like IL-6 and IL-10 and contributing to neuroinflammation. In addition, these RNA processing failures affect how genes are normally regulated at multiple levels, including splicing and miRNA production. When these systems are disrupted, many essential neuronal genes are incorrectly processed, weakening synaptic function and overall neuron stability. Other ALS-related changes, such as C9orf72 repeat expansions, worsen this process by trapping RNA-binding proteins and increasing cellular stress. Over time, the combination of RNA misprocessing, toxic aggregate formation, and chronic inflammatory signaling creates a self-reinforcing cycle in which RNA damage triggers inflammation and inflammation accelerates RNA dysfunction [64].

### 4.7. Alzheimer’s Disease

AD is a progressive neurodegenerative disorder characterized by a gradual decline in memory and cognition, ultimately leading to loss of independence. It accounts for roughly two-thirds of dementia cases in individuals aged 65 years and older. Two hallmark pathologies drive disease progression: accumulation of neurotoxic amyloid-β (Aβ), which contributes to neuronal dysfunction, and aggregation of hyperphosphorylated tau into insoluble neurofibrillary tangles that disrupt synaptic signaling [65]. The progressive formation of neuritic plaques and tangles is accompanied by pathological neuroinflammation, a central contributor to AD and many other CNS disorders [66,67].

Increasing evidence indicates that ncRNAs are key regulators of neuroinflammatory signaling. Notably, specific ncRNAs may be shared across multiple inflammatory and neurodegenerative conditions, suggesting common regulatory circuits. Within these networks, lncRNAs interact extensively with other RNA species: they can function as miRNA sponges to modulate miRNA availability, while miRNAs can also influence lncRNA expression, activation, and stability. Because lncRNAs are highly expressed in mammalian neural tissues, they are well-positioned to respond rapidly to environmental and molecular stressors, and they have been implicated in AD, PD, and Huntington’s disease [68]. One example is MALAT1, which has been reported to suppress neuronal apoptosis and reduce neuroinflammation in AD models [69]. Proposed mechanisms include attenuation of microglial activation through NF-κB inhibition and suppression of miR-125b. In AD models where MALAT1 is overexpressed, miR-125b levels decrease, which is associated with reduced inflammatory cytokine release [70].

In parallel, miRNAs, which are small single-stranded ncRNAs (~19–25 nucleotides), post-transcriptionally regulate gene expression by binding complementary sequences in the 3′UTR of target mRNAs [71]. Many miRNAs are enriched in immune cells and can modulate their activation and function. miRNAs such as miR-155, miR-146, and miR-223 have been linked to TLR-associated inflammatory regulation and are widely implicated in neuroinflammatory responses [72]. Importantly, transcription of several inflammation-related miRNAs is regulated by NF-κB, a stress- and immune-induced transcription factor. Under physiological conditions, acetylcholine can dampen inflammation-induced NF-κB activation, providing an additional layer of control that intersects with miRNA-mediated regulation of neuroinflammation [73].

In summary, the RNA–Inflammation Axis describes a hierarchical signaling network in which dysregulated coding and non-coding RNAs function as upstream drivers of inflammation. These RNAs engage RNA-sensing machinery or gene-regulatory programs, activate core inflammatory pathways, and produce disease-specific cellular and tissue outcomes, each of which represents a potential target for RNA-based therapeutic intervention (Table 1).

## 5. Inter-Organ Crosstalk: A Novel Perspective

Emerging evidence suggests that inflammatory RNA signaling is not confined within individual organs but instead operates through a dynamic, systemic communication network. In this context, the concept of a gut–brain–tumor axis provides a useful framework to understand how peripheral inflammation, RNA signaling, and neurodegeneration converge. A key mediator of this inter-organ dialogue is extracellular vesicles, particularly exosomes, which function as mobile carriers of regulatory RNA species across biological barriers.

### 5.1. The Gut–Brain–Tumor Triangle

The gut–brain axis has long been recognized as a bidirectional communication system integrating neural, immune, and metabolic pathways. More recent studies extend this concept to a broader gut–brain-tumor triangle, where peripheral tumors and gut-derived signals influence central nervous system (CNS) inflammation through RNA-mediated mechanisms. Extracellular vesicles (EVs), enriched with microRNAs and other non-coding RNAs, have emerged as critical mediators of this communication network [43,82]. Gut microbiota and intestinal epithelial cells release EVs carrying regulatory RNA cargo that reflects the inflammatory or dysbiotic state of the host. These vesicles can enter systemic circulation and, importantly, have been shown to cross the blood–brain barrier (BBB), particularly under pathological conditions where barrier integrity is compromised [83,84]. Once in the CNS, these RNA-containing vesicles can modulate neuronal and glial gene expression, thereby influencing neuroimmune responses. In parallel, peripheral tumors contribute to this axis by releasing inflammatory RNAs either freely or within vesicles. Chronic systemic inflammation, common in cancer, can weaken BBB integrity, facilitating the entry of circulating RNA species and vesicles into the brain. Studies indicate that inflammatory conditions enhance vesicle trafficking across the BBB, likely through increased transcytosis and barrier permeability [85]. Functionally, these RNA signals can activate microglia and astrocytes, triggering pathways such as NF-κB and MAPK that sustain neuroinflammation. For example, gut- or immune-derived exosomes enriched in pro-inflammatory microRNAs and pathogen-associated molecules can engage TLRs in the brain, leading to persistent glial activation and cytokine release [86]. This provides a mechanistic link between peripheral inflammation, whether driven by gut dysbiosis or tumor burden, and central neurodegenerative processes. Taken together, the triangle model highlights a key shift, such as inflammatory RNAs are not merely local regulators but systemic messengers capable of reshaping distant tissue environments, particularly when physiological barriers are compromised.

### 5.2. Exosomal Mail: Systemic RNA Messaging in Disease Progression

Exosomes can be viewed as a biological mail system, selectively packaging and delivering RNA cargo to distant cells. Tumor-derived exosomes are especially relevant in this context, as they are enriched with oncogenic and inflammatory non-coding RNAs, including miRNAs, lncRNAs, and circRNAs. These vesicles play a central role in preparing distant tissues for disease progression. In cancer, exosomal RNAs contribute to the formation of pre-metastatic niches by reprogramming recipient cells in distant organs. For instance, tumor-derived exosomal non-coding RNAs have been shown to regulate angiogenesis, immune evasion, and stromal remodeling hallmarks essential for metastasis [87]. This priming effect extends beyond classical metastatic sites and may include the brain, particularly in cancers associated with cognitive impairment or brain metastases. Importantly, exosomes can cross the BBB and deliver functional RNA cargo to neural cells. Experimental studies demonstrate that systemically administered exosomes can transfer small interfering RNAs (siRNAs) or endogenous regulatory RNAs to neurons, microglia, and oligodendrocytes, leading to measurable changes in gene expression within the CNS [88]. This supports the idea that tumor-derived exosomes in circulation can directly influence brain biology. From a neurodegenerative perspective, this exosomal communication has profound implications. Peripheral exosomes carrying pro-inflammatory RNAs can prime the brain by inducing a sustained inflammatory phenotype in glial cells. This includes enhanced microglial activation, increased production of cytokines such as TNF-α, and elevated oxidative stress all of which contribute to synaptic dysfunction and neuronal loss [86]. An additional layer of complexity arises from the selective packaging of RNA cargo. Tumor cells actively sort specific non-coding RNAs into exosomes, suggesting a regulated process aimed at modifying distant microenvironments. In glioma and other cancers, exosomal RNAs have been shown to promote proliferation, invasion, and immune escape in recipient cells, underscoring their role as functional signaling entities rather than passive byproducts [89]. Specific exosomal ncRNAs have emerged as important mediators of intercellular inflammatory communication. Tumor-derived exosomes enriched in miR-21, miR-155, and lncRNA HOTAIR promote macrophage polarization and metastatic niche formation, whereas neuron- and glia-derived exosomes carrying miR-124, miR-146a, and miR-21 regulate microglial activation and neuroinflammatory responses [90,91].

Inter-organ crosstalk mediated by inflammatory RNAs represents a paradigm shift in our understanding of disease biology. The gut–brain-tumor triangle and exosomal RNA transfer illustrate how local pathological signals can propagate systemically, bridging cancer and neurodegeneration. These insights not only deepen our understanding of disease mechanisms but also open new avenues for therapeutic intervention, particularly through targeting exosomal communication and RNA trafficking across biological barriers.

## 6. Shared RNA-Inflammatory Mechanisms in Cancer and Neurodegeneration

Although cancer and neurodegenerative diseases are traditionally viewed as distinct pathological entities, growing evidence suggests that they share several fundamental RNA-mediated inflammatory mechanisms. At the center of both conditions is a chronic inflammatory state sustained by complex interactions between ncRNAs, innate immune receptors, and inflammatory signaling pathways. Dysregulated miRNAs, lncRNAs, and circRNAs modulate key inflammatory regulators such as NF-κB, STAT3, MAPK, and inflammasome signaling, thereby influencing cell survival, proliferation, immune activation, and tissue remodeling [1,92]. Persistent activation of these pathways contributes to tumor growth and immune evasion in cancer while promoting glial activation, neuronal dysfunction, and progressive neurodegeneration in disorders such as AD and PD [93,94].

Several ncRNAs have emerged as common regulators across both disease classes. For example, miR-21 and miR-155 are frequently upregulated in tumors and neuroinflammatory conditions, where they enhance pro-inflammatory cytokine production and activate NF-κB-dependent signaling. In contrast, miR-146a generally functions as a negative regulator of inflammation by suppressing TLR and cytokine signaling pathways, although its expression is often dysregulated during disease progression [95,96]. These findings suggest that shared ncRNA networks may serve as central modulators of chronic inflammation regardless of tissue type.

Another important convergence point is the role of extracellular vesicles, including exosomes, in intercellular communication. Both cancer cells and activated neural cells release exosomes enriched with inflammatory RNAs, miRNAs, and other signaling molecules capable of altering the behavior of recipient cells. Tumor-derived exosomes can promote metastasis and immune suppression, whereas exosomal RNA signaling within the central nervous system contributes to microglial activation and neuroinflammation [71,90]. Furthermore, RNA-sensing receptors, including TLR3, TLR7, TLR8, and RIG-I-like receptors, recognize endogenous and exogenous RNA species and trigger inflammatory cascades that are implicated in both tumor progression and neurodegenerative pathology [97]. Collectively, these observations support the concept that cancer and neurodegeneration are linked by a common RNA–inflammation network in which dysregulated RNA signaling perpetuates chronic inflammation and disease progression. Understanding these shared mechanisms may facilitate the development of RNA-based therapeutic strategies with broad applicability across multiple inflammatory diseases.

## 7. RNAi and Silencing Technologies

RNAi is a powerful gene-silencing mechanism in which short interfering RNAs (siRNAs) guide the degradation of complementary mRNA targets. As a therapeutic strategy, RNAi offers a major advantage over many small molecules because, in principle, nearly any gene can be targeted through sequence complementarity. What began as a bench technique has now evolved into a clinically validated platform, with expanding modalities and increasingly sophisticated designs that extend well beyond first-generation siRNAs.

**Figure 2:**
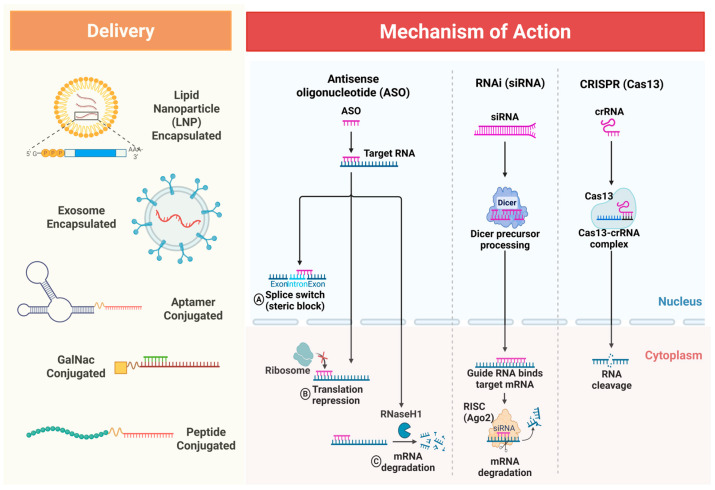
Delivery platforms and mechanisms of action of RNA-based therapeutics: RNA-based therapeutics are administered through a range of delivery platforms (**left panel**) including lipid nanoparticles (LNPs)/liposomes, exosome-based carriers, and chemical conjugates (aptamer, GalNAc, and peptide) that facilitate tissue-specific targeting and cellular uptake. Following intracellular delivery, RNA therapeutics exert their effects through three primary mechanisms (**right panel**). Antisense oligonucleotides (ASOs) act via Ⓐ splice switching through steric blocking of pre-mRNA splice sites in the nucleus, Ⓑ translational repression through ribosome steric blockade in the cytoplasm, or Ⓒ RNase H1-mediated degradation of the target RNA:DNA hybrid in the cytoplasm. RNA interference (RNAi) via synthetic siRNA duplexes loads directly into the RNA-induced silencing complex (RISC) through the AGO2 protein, which directs guide-strand-mediated recognition and cleavage of complementary target mRNA sequences. CRISPR-Cas13 utilizes a CRISPR RNA (crRNA) to guide the Cas13 effector protein to complementary RNA targets in the cytoplasm, enabling programmable RNA cleavage without DNA editing. **Abbreviations:** RISC: RNA-induced silencing complex; AGO2: Argonaute-2; CIRSPR: Clustered Regularly Interspaced Short Palindromic Repeats; Cas-13: CRISPR-associated protein 13.

Next-generation siRNA development has focused on improving durability, specificity, and delivery. Chemical modifications such as 2′-O-methyl, 2′-fluoro, and phosphorothioate linkages, along with strand engineering and optimized thermodynamics, enhance stability while reducing off-target activity and innate immune stimulation. Delivery innovations have been equally transformative (Figure 2). In particular, ligand conjugation has enabled potent, durable gene silencing with convenient subcutaneous dosing schedules that can extend from monthly to even twice-yearly administration in some programs [98]. As a result, the clinical trajectory of RNAi therapeutics illustrates a central translational lesson: delivery chemistry often determines clinical feasibility. Patisiran, formulated in lipid nanoparticles (LNPs), demonstrated clinically meaningful improvement in neuropathy in hereditary transthyretin-mediated amyloidosis, establishing intravenous LNP delivery as an effective approach for liver-directed siRNA therapy (Table 2) [99]. Building on this success, GalNAc-conjugated siRNAs demonstrated that receptor-mediated uptake can achieve similarly strong hepatic silencing while offering improved dosing convenience. Vutrisiran, approved in 2022, targets the same pathway as patisiran but leverages enhanced stabilization chemistry and GalNAc conjugation to enable lower-dose, subcutaneous administration [100].

Antisense oligonucleotides (ASOs) offer a complementary approach to silencing with distinct advantages. One of the primary mechanisms by which ASOs exert their effects is through interaction with ribonuclease H (RNase H), an endogenous enzyme that cleaves the RNA strand in RNA-DNA hybrid regions (Figure 2). ASOs designed to leverage this interaction typically consist of a central DNA region flanked by chemically modified nucleotides, a structure known as a ‘gapmer’. This configuration enables the ASO to effectively engage RNase H and promote targeted RNA degradation [101]. Another crucial mechanism of ASO action involves modulating RNA splicing. By targeting specific RNA sequences involved in splicing, such as splice sites or regulatory elements, ASOs can alter the splicing patterns of target genes. This approach has proven particularly effective in treating genetic disorders caused by splicing defects, with nusinersen (Spinraza), an FDA-approved drug for spinal muscular atrophy, serving as a prime example of this therapeutic strategy (Table 2). ASOs have been especially impactful in the CNS because intrathecal delivery is clinically practical and because splicing modulation can rescue protein function rather than just reducing expression [102].

A major emerging direction in RNA therapeutics is RNA-targeting CRISPR systems, particularly Cas13, which cleaves RNA rather than DNA. Cas13 enables programmable RNA knockdown (Figure 2) and, in catalytically inactive forms, can be coupled to editing domains to support targeted RNA modification [103]. Therapeutically, this approach is attractive because it is reversible (no permanent genome edits) and allows dynamic control of transcript levels, offering a potentially safer alternative to DNA-targeting nucleases such as Cas9 for inducing loss-of-function phenotypes without altering the genome [104]. Cas13 family effectors (Cas13a–Cas13d) can efficiently cleave complementary single-stranded RNA targets, but key challenges remain, including the delivery of larger ribonucleoprotein complexes, the management of collateral activity (which varies by Cas13 variant), and the achievement of predictable on-target behavior in vivo [105].

At the same time, the integration of artificial intelligence (AI) with molecular biology is accelerating advances in RNA silencing and genome editing technologies. Cas13, therapeutic performance depends on a complex interplay of factors, including guided RNA sequence, RNA secondary structure, target accessibility, context dependence, and intrinsic properties of the Cas13 enzyme itself. Because these variables are highly interconnected, AI-based models are becoming increasingly valuable for predicting efficacy and reducing unintended effects. More broadly, AI is reshaping RNA therapeutics in three major ways: enhancing sequence design to maximize potency and minimizing off-target binding; accelerating chemistry and formulation decisions by identifying modification patterns and delivery compositions that perform best in specific tissues; and enabling structure- and interaction-based modeling to reduce immunostimulation and toxicity risks by anticipating innate sensing and unintended interactions with RNA-binding proteins [106].

Overall, the field is moving toward smarter molecular designs, improved delivery beyond the liver and CNS, and integrated workflows, in which AI helps optimize both the RNA therapeutic construct and the delivery system, which ultimately determines whether silencing is achieved in the intended cells.

## 8. Targeted Delivery

A central challenge in translating RNA-based therapeutics for cancer and neurodegeneration lies not in molecular design, but in delivery. The inflammatory RNA landscape comprising miRNAs, lncRNAs, and other regulatory species demands precise spatial control. This is particularly critical in neurodegenerative diseases, where therapeutic molecules must cross the highly restrictive blood–brain barrier (BBB), and in cancer, where inflamed and heterogeneous microenvironments complicate targeting.

### 8.1. Overcoming the BBB

The BBB is a tightly regulated interface formed by endothelial cells with strong junctional complexes, supported by pericytes and astrocytic end feet. While essential for maintaining neural homeostasis, it severely limits the entry of most therapeutic agents, especially large or hydrophilic molecules such as RNA therapeutics [107]. Consequently, inefficient BBB penetration remains a major cause of failure in CNS drug development [108]. To address this, nanotechnology-based delivery systems have been engineered to exploit both passive and active transport mechanisms. Nanocarriers can facilitate transcytosis across endothelial cells, protect RNA cargo from enzymatic degradation, and enable controlled release within the CNS [109]. Strategies such as receptor-mediated transport (e.g., via transferrin or insulin receptors), adsorption-mediated transcytosis, and transient BBB modulation, including focused ultrasound or permeation enhancers, have shown promise in improving delivery efficiency [110]. Importantly, the success of RNA therapeutics in non-CNS diseases underscores their potential. However, achieving similar outcomes in neurodegeneration requires overcoming this delivery bottleneck through multidisciplinary innovation in nanocarrier design and targeting strategies [110].

### 8.2. Biomimetic Nanocarriers

Biomimetic nanocarriers represent a significant conceptual shift in drug delivery, moving from purely synthetic systems to hybrid platforms that emulate biological structures. These systems, often coated with natural cell membranes derived from erythrocytes, immune cells, platelets, or even cancer cells, inherit functional proteins and surface markers that enable immune evasion and prolonged circulation [111]. Such camouflaged nanoparticles can interact more naturally with the host environment, reducing clearance by the mononuclear phagocyte system and enhancing accumulation at sites of inflammation or tumor growth. In the context of the RNA–inflammation axis, this is particularly relevant, as inflamed tissues exhibit specific adhesion molecules and chemokine gradients that biomimetic systems can exploit for homing [112]. Additionally, these carriers can mimic the intrinsic targeting behavior of their source cells. For example, immune cell membrane-coated nanoparticles can preferentially localize to inflammatory niches, while cancer cell membrane coatings enable homotypic targeting through tumor–tumor recognition mechanisms. This dual functionality, immune evasion and active targeting, positions biomimetic nanocarriers as powerful vehicles for delivering RNA therapeutics with improved specificity and reduced systemic toxicity [113].

### 8.3. Ligand-Directed Targeting

Beyond biomimicry, ligand-directed targeting provides an additional layer of precision by functionalizing nanocarriers with molecules that bind selectively to receptors on target cells. These ligands include peptides, antibodies, aptamers, and small molecules, each designed to engage specific cellular markers. In brain-targeted delivery, ligands such as transferrin, apolipoprotein E mimetics, or rabies virus glycoprotein (RVG)-derived peptides facilitate receptor-mediated transport across the BBB and enhance uptake by neurons or glial cells [114]. Similarly, in cancer, ligands targeting overexpressed receptors such as integrins or growth factor receptors enable selective accumulation within tumor tissues. This strategy not only improves delivery efficiency but also reduces off-target effects, a critical consideration for RNA-based therapies that can broadly modulate gene expression. However, challenges remain, including ligand stability, potential immunogenicity, and unintended accumulation in peripheral organs, highlighting the need for continued optimization [114].

In summary, targeted delivery strategies are evolving toward increasingly sophisticated, multi-functional systems that integrate BBB navigation, biomimicry, and ligand specificity. These advances are essential for unlocking the therapeutic potential of RNA silencing approaches, particularly in diseases where inflammation and tissue-specific pathology intersect, such as cancer-associated neurodegeneration.

## 9. Preclinical & Clinical Landscapes

Many RNA therapeutics are currently being developed and clinically tested by pharma and biotech companies worldwide. Modalities such as antisense oligonucleotides (ASOs), siRNA, miRNA modulators, mRNA therapeutics, aptamers, shRNA, and CRISPR/Cas-guided single-guide RNAs are rapidly expanding, driven by advances in high-throughput sequencing, structural biology, and delivery technologies (Table 2).

## 10. Challenges and Limitations

Despite rapid advances in understanding the RNA–inflammation axis, several key challenges continue to limit its full translational potential. One major issue is the context-dependent behavior of ncRNAs. The same RNA molecule can function as either a tumor suppressor or an oncogene depending on cell type, disease stage, and microenvironment. This duality complicates therapeutic targeting, as modulation of a single ncRNA may produce unintended effects in non-target tissues or parallel pathways [131]. Another important limitation lies in the complexity and redundancy of inflammatory signaling networks. Pathways such as NF-κB, STAT3, and MAPK are highly interconnected, and ncRNAs often participate in multiple feedback loops within these systems [132]. As a result, targeting one component may not be sufficient to achieve durable therapeutic effects, and compensatory mechanisms can diminish treatment efficacy. A critical translational barrier is delivery, particularly for RNA-based therapeutics. Efficient and specific delivery to target tissues, especially across the BBB in neurodegenerative diseases, remains challenging. Although nanocarriers and exosome-based systems show promise, issues related to stability, immune clearance, off-target accumulation, and large-scale reproducibility persist. In addition, there is still an incomplete understanding of inter-organ RNA communication, including the dynamics of exosomal RNA transfer between tumors, peripheral organs, and the brain [133]. While growing evidence supports systemic RNA signaling, the precise mechanisms governing RNA packaging, release, targeting, and functional integration in recipient cells remain poorly defined. From a clinical perspective, standardization and validation of RNA biomarkers remain limited. Variability in sample collection, processing, and detection methods can lead to inconsistent results across studies. Furthermore, many findings are derived from preclinical models, and their translation to human disease is not always straightforward.

Despite encouraging advances in RNA-based therapeutics, several challenges have limited successful clinical translation. Many candidate ncRNA biomarkers identified in preclinical studies fail to demonstrate consistent performance across independent patient cohorts due to disease heterogeneity, sample variability, and differences in analytical platforms. Similarly, although numerous miRNA- and siRNA-based therapies have shown efficacy in experimental models, clinical development has been hindered by issues related to off-target effects, immune activation, poor tissue specificity, and inefficient delivery, particularly within the central nervous system. Furthermore, the complex and context-dependent functions of ncRNAs can result in unpredictable biological outcomes, as individual RNA molecules often regulate multiple signaling pathways simultaneously. These limitations underscore the need for improved delivery technologies, standardized biomarker validation pipelines, and a deeper mechanistic understanding of RNA-mediated inflammatory networks before widespread clinical implementation can be achieved [134,135,136].

## 11. Future Direction

Future research in the RNA–inflammation axis should move toward a more integrated, systems-level understanding of RNA-mediated signaling across tissues and disease states. Multi-omics approaches combining transcriptomics, epigenomics, and proteomics will be essential for mapping the dynamic regulatory networks controlled by ncRNAs and identifying key nodes suitable for therapeutic intervention. Advances in precision RNA therapeutics are expected to play a transformative role. Technologies such as siRNA, antisense oligonucleotides, and CRISPR-based RNA editing offer opportunities to selectively modulate disease-associated RNAs. However, their success will depend on improving delivery systems, particularly by developing targeted, biomimetic, and BBB-penetrant nanocarriers that ensure tissue specificity and minimize off-target effects. Another promising direction is the exploration of exosome-based diagnostics and therapeutics. Given their natural role in intercellular communication, engineered exosomes could serve as both biomarkers and delivery vehicles for RNA therapeutics. Understanding how exosomal RNA cargo is selectively packaged and directed to specific tissues will be critical for harnessing this potential.

Importantly, future studies should further investigate the inter-organ crosstalk, particularly within the gut–brain–tumor axis. Deciphering how peripheral inflammation influences central nervous system pathology through RNA signaling may uncover novel therapeutic targets for both cancer-associated neurological complications and primary neurodegenerative diseases. Finally, translating these discoveries into clinical applications will require well-designed clinical trials, standardized methodologies, and interdisciplinary collaboration across molecular biology, bioengineering, and clinical sciences. Bridging these gaps will be essential to move from mechanistic insights to effective therapies.

## 12. Conclusions

Collectively, the evidence reviewed here supports a unifying concept that inflammatory RNAs are not merely biomarkers of disease, but active pathological drivers that can initiate and perpetuate chronic inflammation across cancer and neurodegeneration. Diverse classes of ncRNAs, including miRNAs, lncRNAs, circRNAs, and piRNAs, shape inflammatory tone by controlling transcriptional and post-transcriptional programs, modulating cytokine/chemokine production, and influencing major signaling axes such as NF-κB, STAT3, MAPK, and PI3K/AKT/mTOR. In parallel, innate immune RNA sensors, including endolysosomal TLR3/7/8 and cytosolic RNA recognition systems, translate RNA-associated PAMP/DAMP signals into inflammatory effector programs. When these sensing and regulatory layers fail to resolve, feedback loops emerge in which inflammation drives further RNA dysregulation (e.g., altered processing, editing, or stability), and RNA dysregulation reinforces sustained inflammatory signaling, producing persistent pathology. RNA interference pathways and RNA-guided chromatin remodeling complexes demonstrate how small RNAs can direct histone modifications and heterochromatin formation, creating long-lived transcriptional repression or activation programs that sustain inflammatory states. In cancer, these circuits amplify pro-tumorigenic inflammation and remodel the tumor microenvironment to support survival, invasion, EMT, angiogenesis, and immune evasion; in neurodegenerative disorders, similar ncRNA-driven networks reinforce microglial/astrocytic activation, oxidative stress, and neuronal injury, accelerating disease progression. Thus, the RNA–inflammation axis provides a mechanistic bridge between genetic susceptibility, environmental triggers, and chronic inflammatory disease trajectories. The review also highlights a growing need to view RNA pathology through the lens of inter-organ crosstalk, including the proposed “gut–brain–tumor” triangle and the role of extracellular vesicles as “exosomal mail” that transports inflammatory RNA cargo between tissues. Such long-range signaling may help explain how peripheral inflammation or tumors can contribute to distant immune priming, metastasis, and cognitive decline, particularly when barrier functions are compromised. Recognizing these systemic connections will be critical for identifying where to intervene, whether at the source of inflammatory RNA production, during transit (EV release/uptake), or at target-site sensing and amplification nodes. Collectively, despite their distinct clinical manifestations, cancer and neurodegenerative disorders share common RNA-mediated inflammatory circuits involving NF-κB, STAT3, MAPK, and inflammasome signaling. These interconnected networks, regulated by disease-specific and shared ncRNAs, provide a mechanistic framework for understanding chronic inflammation and represent promising targets for future RNA-based therapeutic interventions.

Finally, the therapeutic landscape is rapidly advancing from conceptual innovation toward clinical translation. RNAi and antisense platforms, emerging RNA editors such as Cas13-based systems, and AI-enabled design-build-test-learn cycles are expanding the precision, adaptability, and durability of transcript-level interventions. Nevertheless, delivery remains the dominant bottleneck, especially beyond the liver and CNS. Future progress will depend on integrating mechanistic insight into engineering solutions, BBB-penetrant and ligand-directed carriers, biomimetic nanoplatforms, and rational selection of payload chemistry to achieve tissue-selective silencing with acceptable safety. As clinical and preclinical pipelines mature, the field is inclined to move from broadly suppressing inflammation to selectively silencing the disease-driving RNA nodes and feedback loops that sustain cancer progression and neurodegeneration, enabling more durable, mechanism-based therapies.

## Figures and Tables

**Table 1:** Representative ncRNAs and inflammatory signaling pathways in cancer and neurodegenerative diseases.

Disease	ncRNA	Major Target/Pathway	Effect
**Breast cancer**	miR-155	IL-6/STAT3, NF-κB	Pro-inflammatory, tumor-promoting [74,75]
**Breast cancer**	NKILA	NF-κB	Anti-inflammatory, tumor suppressive [76]
**Glioblastoma**	MALAT1	PI3K/Akt, NF-κB	Proliferation, inflammation [77]
**Lung cancer**	miR-21	NF-κB, STAT3	Cytokine production, EMT [55]
**Alzheimer’s disease**	miR-146a	TLR/NF-κB	Negative feedback regulator [78]
**Parkinson’s disease**	SNHG1	miR-7/NLRP3/NF-κB	Microglial activation [63]
**Parkinson’s disease**	lncRNA-p21	miR-181/PKC-δ/NF-κB	Neuroinflammation [79]
**ALS**	TDP-43-associated RNAs	Cytokine signaling	Chronic neuroinflammation [80]
**Multiple diseases**	miR-21	NF-κB, STAT3	Shared inflammatory mechanism [81]
**Multiple diseases**	miR-155	NF-κB, MAPK	Persistent inflammation [75]

**Abbreviations:** ncRNA: non-coding RNA; miR: microRNA; IL-6: Interleukin-6; STAT3: signal transducer and activator of transcription; NF-κB: Nuclear Factor kappa-light-chain-enhancer of activated B cells; NKILA: Nuclear Factor-Kappa B interacting long non-coding RNA; MALAT1: Metastasis associated lung adenocarcinoma transcript 1; SNHG1: Small nucleolar RNA host gene 1; TLR: Toll-Like Receptor; NLRP3: NLR family pyrin domain containing 3; lncRNA: Long non-coding RNA; PKC-δ: Protein Kinase C-delta; MAPK: Mitogen-activated protein kinase.

**Table 2:** Clinical-Stage RNA Therapeutics for Cancer and Neurodegenerative Diseases.

Sr. No.	Drug Name	RNA Category	Target/Mechanism	Developer/ Company	Disease	Clinical Stage	Reference
**CANCER**							
**1.**	BNT113	mRNA vaccine (LPX)	HPV16 E6/E7 antigens (immune priming)	BioNTech	HPV16+ recurrent/metastatic HNSCC	Phase II/III	[115]
**2.**	mRNA-2752	mRNA immunotherapy (intratumoral)	Cytokine/immune activation program (intratumoral)	Moderna	Solid tumors (injectable lesions)	Phase I	[116]
**3.**	BNT116	mRNA vaccine	NSCLC tumor-associated antigens	BioNTech	NSCLC	Phase I	[117]
**4.**	V940 (mRNA-4157)	Personalized mRNA vaccine	Patient-specific neoantigens	Moderna/Merck	Melanoma (adjuvant); other solid tumors	Phase III	[118]
**5.**	BNT111	mRNA vaccine (LPX)	Fixed set of melanoma antigens (FixVac)	BioNTech	Melanoma	Phase II	[119]
**6.**	Autogene cevumeran (BNT122/RO7198457)	Personalized mRNA vaccine (LPX)	Individualized neoantigens	BioNTech/Genentech (Roche)	Resected pancreatic cancer; other settings	Phase I	[120]
**7.**	ARO-HIF2 (zifcasiran)	siRNA	HIF-2α mRNA silencing	Arrowhead	Clear cell RCC	Phase I	[121]
**8.**	STP707	siRNA	Dual knockdown: TGF-β1 + COX-2	Sirnaomics	Pancreatic cancer cohorts/solid tumors	Phase I	ClinicalTrials.gov NCT05037149
**9.**	Danvatirsen (AZD9150)	ASO	STAT3 mRNA inhibition	AstraZeneca/Ionis	Solid tumors (multiple)	Phase II	[122]
**10.**	MEDI1191	mRNA (intratumoral, LNP)	IL-12 expression in tumor micro-environment	AstraZeneca/MedImmune	Advanced solid tumors (injectable lesions)	Phase I	[123]
**11.**	STP705	siRNA	Dual knockdown: TGF-β1 + COX-2	Sirnaomics	cSCC in situ/skin cancers	Phase II/IIb	ClinicalTrials.gov NCT04669808
**NEURODEGENERATION**							
**1.**	IONIS-MAPTRx (BIIB080)	ASO	MAPT (tau) mRNA	Ionis/Biogen	Alzheimer’s Disease	Phase II	[124]
**2.**	IONIS-SOD1Rx (Tofersen)	ASO	SOD1 mRNA	Ionis/Biogen	ALS (SOD1)	FDA Approved 2023	[125]
**3.**	Nusinersen (Spinraza)	ASO	SMN2 splicing	Ionis/Biogen	Spinal Muscular Atrophy	FDA Approved 2016	[126]
**4.**	WVE-003	ASO	HTT mRNA (allele-selective)	Wave Life Sciences	Huntington’s Disease	Phase I/II	ClinicalTrials.gov NCT04617860
**5.**	Eplontersen	ASO	TTR mRNA	Ionis/AstraZeneca	hATTR Polyneuropathy	FDA Approved 2023	[127]
**6.**	Patisiran (ONPATTRO)	siRNA (LNP)	TTR mRNA	Alnylam Pharmaceuticals	hATTR Amyloidosis	FDA Approved 2018	[128]
**7.**	Vutrisiran (AMVUTTRA)	siRNA (GalNAc)	TTR mRNA	Alnylam Pharmaceuticals	hATTR Polyneuropathy	FDA Approved 2022	[129]
**8.**	Ulefnersen (ION363)	ASO	FUS mRNA	Ionis Therapeutics	ALS (FUS)	Phase I	[130]

**Abbreviation:** mRNA: messenger RNA; HPV: Human Papillomavirus type 16; HNSCC: Head and Neck Squamous Cell Carcinoma; NSCLC: Non-Small Cell Lung Cancer; LPX: lipoplex; siRNA: Small interfering RNA; HIF-2α: Hypoxia-inducible factor 2-alpha; RCC: Renal cell carcinoma; TGF-β1: Transforming Growth Factor beta 1; COX-2: Cyclooxygenase-2; ASO: Antisense Oligonucleotides; STAT3: Signal Transducer and Activator of Transcription 3; IL-12: Interleukin 12; cSCC: Cutaneous Squamous Cell Carcinoma; MAPT: Microtubule-Associated Protein Tau gene; SOD1: Superoxide Dismutase 1; ALS: Amyotrophic lateral sclerosis; SMN2: Survival Motor Neuron 2; TTR: Transthyretin; hATTR: Hereditary transthyretin amyloidosis; FUS: Fused in Sarcoma.

## Data Availability

No new data were created or analyzed in this study.
